# Association between whole blood ratio and risk of mortality in massively transfused trauma patients: retrospective cohort study

**DOI:** 10.1186/s13054-024-05041-8

**Published:** 2024-07-19

**Authors:** Makoto Aoki, Toshikazu Abe, Akira Komori, Morihiro Katsura, Kazuhide Matsushima

**Affiliations:** 1 Advanced Medical Emergency Department and Critical Care Center, Japan Red Cross Maebashi Hospital, Maebashi, Japan; 2 Division of Traumatology, National Defense Medical College Research Institute, Tokorozawa, Japan; 3 Department of Emergency and Critical Care Medicine, Tsukuba Memorial Hospital, Tsukuba, Japan; 4 Department of Health Services Research, Faculty of Medicine, University of Tsukuba, Tsukuba, Japan; 5 Division of Acute Care Surgery, Department of Surgery, University of Southern California, Los Angeles, USA

**Keywords:** Whole blood, Massive blood transfusion, Ratio, Mortality, Trauma

## Abstract

**Background:**

Although whole blood (WB) transfusion was reported to improve survival in trauma patients with hemorrhagic shock, little is known whether a higher proportion of WB is associated with an improved survival. This study aimed to evaluate the association between whole blood ratio (WBR) and the risk of mortality in trauma patients requiring massive blood transfusion.

**Methods:**

We performed a retrospective cohort study from the ACS-TQIP between 2020 and 2021. Patients were aged ≥ 18 years and received WB within 4 h of hospital arrival as a part of massive blood transfusion. Study patients were categorized into four groups based on the quartiles of WBR. Primary outcome was 24-h mortality and secondary outcome was 30-day mortality. Multivariable logistic regression analysis, fitted with generalized estimating equations, was performed to adjust for confounding factors and accounted for within-hospital clustering.

**Results:**

A total of 4087 patients were eligible for analysis. The median age was 37 years (interquartile range [IQR]: 27–53 years), and 85.0% of patients were male. The median number of WB transfusions was 2.3 units (IQR 2.0–4.0 units), and the total transfusion volume was 4940 ml (IQR 3350–8504). When compared to the lowest WBR quartile, the highest WBR quartile had lower adjusted 24-h mortality (adjusted odds ratio [AOR]: 0.61, 95% confidence interval [CI]: 0.46–0.81) and 30-day mortality (AOR 0.58; 95% CI 0.45–0.75).

**Conclusion:**

The probability of mortality consistently decreased with higher WBR in trauma patients requiring massive blood transfusion.

**Supplementary Information:**

The online version contains supplementary material available at 10.1186/s13054-024-05041-8.

## Introduction

Hemorrhage remains a leading cause of preventable death in injured civilian and military patients [1, 2]. The adoption of damage control resuscitation in recent years has contributed to decreased mortality associated with hemorrhagic shock [3]. Blood component therapy involves transfusion of blood components in a balanced ratio to replace blood loss, approximating reconstituted whole blood (WB).

Recently, the use of cold-stored whole blood (WB) was introduced for the management of civilian trauma patients in the United States. Previous literatures reported the potential survival benefit by the use of WB, particularly in patients with hemorrhagic shock [4–7]. However, it remains unknown whether more liberal use of WB transfusion is associated with improved survival. In previous studies evaluating the use of WB in trauma patients with hemorrhagic shock, the amount of WB administered during the initial 24-h period typically ranged 1–2 units even in patients requiring massive blood transfusion [4–6]. In an ideal situation, a total transfusion with WB is desirable for massively transfused patients; however, the use of WB depends on each hospital’s practice due to limited resources.

Hence, we conducted this study to assess the association between a proportion of WB and mortality in massively transfused patients. We calculated whole blood ratio (WBR) by dividing the number of WB units by the sum of WB units and PRBCs units and hypothesized that increased WBR would be associated with improved mortality.

## Methods

### Study design, setting, and data source

This study was a retrospective cohort study using coded data in the American College of Surgeons Trauma Quality Improvement Program (ACS-TQIP) between January 2020 and December 2021. The TQIP database includes a subset of patients from the National Trauma Data Bank (NTDB), who were admitted in American Level 1 or 2 trauma centers, with age > 16 years and abbreviated injury scale (AIS) score > 2 in at least 1 body region. Data were handled in line with the TQIP data-user agreement and access was granted by the ACS TQIP. The Institutional Review Board of the Japan Red Cross Maebashi Hospital deemed the study exempt and waived the need for informed consent from patients (IRB 2021–4), as the data were publicly accessible and de-identified. This study adhered to the Strengthening the Reporting of Observational Studies in Epidemiology (STROBE) guidelines, and a complete checklist has been provided in Supplementary Table 1.

### Study participants

The study comprised patients aged 18 years and older who received WB transfusions within 4 h of hospital arrival as a part of massive blood transfusion. Massive blood transfusion was defined as the administration of 5 units or more of WB or packed red blood cells (PRBCs) within 4 h [8–12]. The following patients were excluded: (1) transfer from another hospital, (2) cardiac arrest upon hospital arrival, (3) Abbreviated Injury Scale (AIS) of 6 in any body region, (4) admitted to the hospital with < 10 cases requiring massive blood transfusion with WB. The number of patients requiring massive blood transfusion at each trauma center is shown in Supplementary Fig. 1.

### Exposure and primary outcome measures

WBR was calculated for each patient by dividing the number of WB units by the sum of WB units and PRBCs units [13]. Our study patients were then categorized into four groups based on the quartiles of WBR. We examined the association between WBR category and outcome. Our primary outcome was 24-h mortality. Secondary outcomes, chosen a priori, included 30-day mortality, total blood transfusion volume (TBV), intensive care unit (ICU) length of stay (LOS), and major complications. Major complications comprised acute kidney injury (AKI), deep vein thrombosis (DVT), acute respiratory distress syndrome (ARDS), stroke, and myocardial infarction (MI).

#### Variables

Patient baseline characteristics included age, sex, injury type, vital signs on hospital arrival, Glasgow coma scale (GCS), injury characteristics including AIS (head, chest, abdomen, extremity) and injury severity score (ISS), transfusion volume including PRBCs, plasma, platelets, and WB within 4 h, timing of whole blood administration, hemorrhage control procedures within 4 h including thoracotomy and laparotomy, hospital information including trauma center level and university affiliation, length of stay, and complications.

#### Statistical analysis

Continuous and categorical variables were presented as medians with interquartile ranges (IQR) and counts with percentages, respectively. Four study groups were compared using the Kruskal–Wallis H-test for continuous variables and the Chi-square test for categorical variables. Missing data were addressed by creating 20 datasets with substituted plausible values through a Markov chain Monte Carlo algorithm, specifically using chained equations imputation [14]. This included imputation for sex (2.0% missing), systolic blood pressure (4.9% missing), heart rate (2.7% missing), GCS (2.1% missing), 24-h mortality (5.4%) and 30-day mortality (5.3%) and trauma center designation (17.5%).

First, we performed the multivariable logistic regression analysis fitted with generalized estimating equations (GEE) adjusting for patient demographics, vital signs on hospital arrival, injury characteristics, hemorrhage control procedures, hospital information, total transfusion volume and timing of WB administration, accounting for within-hospital clustering. The adjusted variables were selected considering clinical knowledge and referencing previous literatures [4, 7, 15, 16]. The WBR category 1 group (first quartile) served as a reference, and WBR category was included as a categorical variable in the multivariable logistic regression model. Two regression models, with and without adjustment for total transfusion volume, were created. Though transfusion volume is thought to be a partial mediator between intervention and outcome, we incorporated TBV into model 2 to adjust trauma severity more rigorously. Besides, we performed the multivariable logistic regression analysis fitted with GEE by adjusting whole blood ratio as continuous variable.

Subsequently, survival curves were estimated using the Kaplan–Meier method, and the log-rank test was employed for comparing between the four WBR categories. The log-rank trend analysis tested the linear trend of the association between WBR category and primary outcomes. We performed an analysis survival with the use of a Cox proportional hazard with adjustment for patient demographics, vital signs on hospital arrival, injury characteristics, hemorrhage control procedures, hospital information, total transfusion volume and timing of WB administration.

Additionally, adjusted event rates were determined by logistic regression based on patient demographics, injury characteristics, vital signs at hospital arrival, hemorrhage control procedures, hospital information, total transfusion volume, timing of WB administration and WBR. In these models, WBR was incorporated as a continuous variable. The comparisons among WBR category were conducted by one way analysis of variance and post hoc analysis of pair-wise t-test with Bonferroni adjustment.

Further analysis included linear and binary multiple variable regression to assess the effect of WBR category on secondary outcomes. Sensitivity analysis was performed by excluding WBR 1 category and using WBR 2 category as a reference for the multivariable logistic regression analysis fitted with GEE. Estimates were calculated with 95% confidence intervals (CIs), and statistical significance was defined as a two-sided *P* value < 0.05. All statistical analyses were performed using R software (version 4.2.2; R Foundation for Statistical Computing, Vienna, Austria).

## Results

**Fig. 1:**
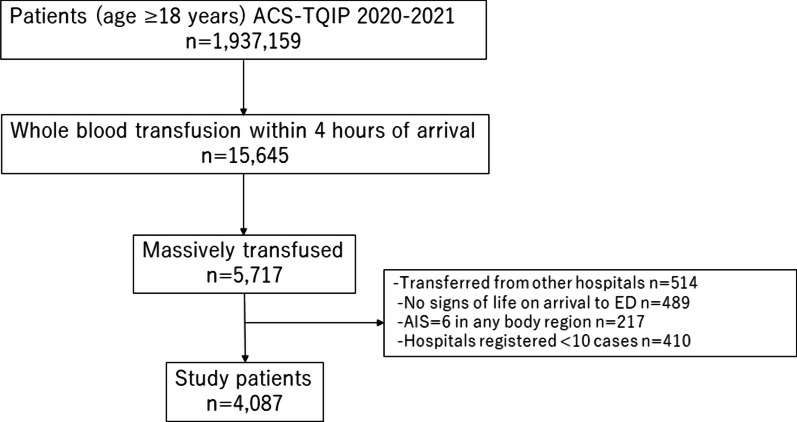
Patient selection flow diagram. *ACS-TQIP* American College of Surgeons Trauma Quality Improvement Program, *ED* emergency department, *AIS* abbreviated injury scale, *WB* whole blood

A total of 5717 patients who received WB as a part of massive blood transfusion were identified. Of those, 4087 patients were included for analysis (Fig. 1). The median age was 36 years (IQR 27–53), and 85.0% were men. The median ISS was 27 (IQR 17–38). The median number of WB transfusion was 2.3 units (IQR 2.0–4.0) and the median TBV was 4940 ml (IQR 3350–8504).

Table 1 shows the characteristics of our study patients by WBR groups. Compared to patients in the WBR2-4 groups, those in the WBR1 group were more likely to sustain penetrating injury and present with hemodynamic instability. TBV down trended from WBR1 to WBR4 (9800 ml in WBR1 to 3500 ml in WBR4). Patients in the WBR1 group more commonly required hemorrhage control procedures (60.9% laparotomy, 15.0% thoracotomy) compared to the other WBR groups. The proportion of patients admitted to Level 1 and/or university-affiliated trauma centers was highest in the WBR4 group. There was no clinically significant difference in ICU LOS between the WBR groups. The incidence of AKI was highest in the WBR1 group and down trended in the WBR2-4 groups.

**Table 1:** Characteristics of studied patients divided into whole blood ratio (WBR) Groups by interquartile range

WBR group	WBR1	WBR2	WBR3	WBR4	*P* value
	0–14.6%	14.6–26.9%	26.9–44.4%	44.4–100%	
	n = 1050	n = 993	n = 1011	n = 1033	
Demographics					
Age, y	35 (26–51)	36 (27–53)	37 (27–54)	37 (27–54)	0.08
Sex, male n (%)	902 (85.9)	852 (85.8)	831 (82.2)	889 (86.1)	0.04
Injury type, n (%)					
Blunt	543 (51.7)	545 (54.9)	598 (59.1)	587 (56.8)	0.01
Penetrating	507 (48.3)	448 (45.1)	412 (40.8)	446 (43.2)	
Vital signs, median (IQR)					
sBP, mmHg	97 (78–122)	98 (80–124)	98 (78–122)	100 (80–124)	0.13
HR, bpm	117 (94–137)	114 (90–133)	114 (94–134)	112 (88–131)	< 0.01
GCS	11 (3–15)	13 (3–15)	13 (3–15)	13 (3–15)	< 0.01
Injury characteristics, median (IQR)					
Head-AIS	3 (2–5)	3 (2–5)	3 (2–4)	3 (2–4)	0.4
Chest-AIS	3 (3–4)	3 (3–4)	3 (3–4)	3 (3–4)	< 0.01
Abdomen-AIS	4 (3–5)	3 (2–4)	3 (2–4)	3 (2–4)	< 0.01
Extremity-AIS	3 (2–4)	3 (2–4)	3 (2–3)	3 (2–3)	< 0.01
ISS, median (IQR)	30 (22–42)	27 (18–38)	26 (17–36)	26 (17–34)	< 0.01
TV, units, median (IQR)					
pRBCs	16.0 (10.5–26.1)	7.0 (5.4–11.9)	5.0 (4.0–7.0)	2.2 (1.0–3.5)	< 0.01
Plasma	12.0 (6.7–20.9)	5.6 (3.6–9.1)	4.0 (2.0–6.0)	1.2 (0–3.0)	< 0.01
Platelets	9.1 (4.1–16.0)	4.6 (0–7.0)	3.6 (0–5.5)	0 (0–3.6)	< 0.01
WB	1.4 (1.0–2.0)	2.0 (1.4–3.0)	2.8 (2.0–4.0)	5.0 (4.0–6.0)	< 0.01
TBV	40.1 (24.5–65.1)	19.4 (13.2–30.6)	15.3 (9.6–22.1)	9.7 (7.0–14.5)	< 0.01
TBV in liters, median (IQR)	9.8 (6.0–15.6)	5.0 (3.6–7.7)	4.1 (3.0–6.1)	3.5 (3.0–4.8)	< 0.01
Timing of WB administration, min	12 (6–23)	13 (7–28)	13 (6–28)	13 (6–31)	0.04
Hemorrhage control procedure, n (%)					
Thoracotomy	157 (15.0)	118 (11.9)	113 (11.2)	107 (10.4)	< 0.01
Laparotomy	639 (60.9)	493 (49.6)	436 (43.1)	442 (42.8)	< 0.01
Hospital information, n (%)					
Trauma center level 1	964 (91.8)	915 (92.1)	874 (86.4)	953 (92.3)	< 0.01
University affiliated	793 (75.5)	732 (73.7)	715 (70.7)	848 (82.1)	< 0.01
ICU LOS in days, median (IQR)	5 (2–15)	6 (3–15)	6 (3–13)	6 (3–13)	< 0.01
Complications*, n (%)					
AKI	120 (16.8)	66 (8.1)	58 (6.7)	48 (5.3)	< 0.01
DVT	67 (9.4)	53 (6.5)	50 (5.8)	86 (9.5)	< 0.01
PE	35 (4.9)	29 (3.6)	40 (4.7)	53 (5.9)	0.16
ARDs	29 (4.1)	22 (2.7)	24 (2.8)	24 (2.7)	0.32
Stroke	24 (3.4)	16 (2.0)	14 (1.6)	22 (2.4)	0.12
MI	8 (1.1)	3 (0.4)	6 (0.7)	5 (0.6)	0.32

*WBR* whole blood ratio, *IQR* interquartile range, *sBP* systolic blood pressure, *HR* heart rate, *GCS* Glasgow coma scale, *AIS* abbreviated injury scale, *ISS* injury severity score, *TV* transfusion volume, *pRBC* packed red blood cell, *WB* whole blood, *TBV* total blood transfusion volume, *ICU* intensive care unit, *LOS* length of hospital stays, *AKI* acute kidney injury, *DVT* deep vein thrombosis, *PE* pulmonary embolism, *ARDs* acute respiratory distress syndrome, *MI* myocardial infarction

*Exclusion of those who died within 24 h

Unadjusted and adjusted study outcomes were shown in Table 2 and Supplementary Table 2. Crude 24-h and 30-day mortality were lowest in the WBR4 group (12.7% and 22.2%, respectively). Compared to the WBR1 group, the adjusted odds of 24-h mortality in the WBR2, WBR3, and WBR4 groups were reduced by 15% (OR 0.85, 95% CI 0.66–1.10), 25% (OR 0.75, 95% CI 0.57–0.98), and 39% (OR 0.61, 95% CI 0.46–0.81), respectively (GEE model 2). Similarly, the adjusted odds for 30-day mortality were reduced by 21% (OR 0.79, 95% CI 0.62–1.01), 27% (OR 0.73, 95% CI 0.57–0.93), and 42% (OR 0.58, 95% CI 0.45–0.75), respectively (GEE model 2). The results of sensitivity analysis (multivariable analysis including only WBR2-4) demonstrated that the WBR3 and WBR 4 groups exhibited lower adjusted odds of 24-h mortality and 30-day mortality compared to the WBR2 group (Supplementary Table 3).

**Table 2:** Mortality outcomes in whole blood ratio groups divided by interquartile range

WBR group	WBR group			
	WBR1	WBR2	WBR3	WBR4
IQR	0–14.6%	14.6–26.9%	26.9–44.4%	44.4–100%
n	n = 1050	n = 993	n = 1011	n = 1033
24-h mortality	336 (32.0)	181 (18.2)	151 (14.9)	131 (12.7)
OR (95% CI)				
Crude	Reference	0.47 (0.38–0.58)	0.37 (0.30–0.46)	0.30 (0.24–0.38)
GEE model 1	Reference	0.51 (0.41–0.65)	0.40 (0.31–0.51)	0.31 (0.24–0.39)
GEE model 2	Reference	0.85 (0.66–1.10)	0.75 (0.57–0.98)	0.61 (0.46–0.81)
30-day mortality	498 (47.4)	296 (29.8)	264 (26.1)	229 (22.2)
OR (95% CI)				
Crude	Reference	0.47 (0.39–0.56)	0.39 (0.32–0.47)	0.31 (0.26–0.38)
GEE model 1	Reference	0.49 (0.39–0.61)	0.40 (0.32–0.49)	0.30 (0.24–0.37)
GEE model 2	Reference	0.79 (0.62–1.01)	0.73 (0.57–0.93)	0.58 (0.45–0.75)

GEE model 1 was adjusted for age, sex, type of penetrating injury, sBP, HR, GCS, AIS for head, chest, abdomen, and peripheral injuries, ISS, timing of WB administration, thoracotomy, laparotomy, trauma center level, and university affiliation

GEE model 2 was adjusted for age, sex, type of penetrating injury, sBP, HR, GCS, AIS for head, chest, abdomen, and peripheral injuries, ISS, timing of WB administration, thoracotomy, laparotomy, trauma center level, university affiliation, and total transfusion volume (pRBCs, plasma, platelets, and WB)

*WBR* whole blood ratio, *IQR* interquartile range, *OR* odds ratio, *CI* confidence interval, *GEE* generalized estimating equation, *sBP* systolic blood pressure, *HR* heart rate, *GCS* Glasgow coma scale, *AIS* abbreviated injury scale, *ISS* injury severity score, *TV* transfusion volume, *pRBC* packed red blood cell, *WB* whole blood

The results of multivariable logistic regression analysis fitted with GEE by adjusting whole blood ratio as continuous variable were shown in Table 3. One percent increment of WBR was not associated with decreased 24-h mortality, however, one percent increment of WBR (including only WBR 2–4) and ten percent increment of WBR were associated with decreased 24-h mortality, (OR 0.99, 95% CI 0.98–0.99) and (OR 0.93, 95% CI 0.89–0.97), respectively.

**Table 3:** Primary outcome in whole blood ratio as continuous variable

Outcomes	OR (95%CI)	*P* value
24-h mortality		
Crude	0.98 (0.97–0.98)	< 0.01
1% increment	0.99 (0.99–1.01)	0.73
1% increment (WBR1 exclusion)	0.99 (0.98–0.99)	< 0.01
10% increment	0.93 (0.89–0.97)	< 0.01

The models were adjusted for age, sex, type of penetrating injury, sBP, HR, GCS, AIS for head, chest, abdomen, and peripheral injuries, ISS, timing of WB administration, thoracotomy, laparotomy, trauma center level, university affiliation, and total transfusion volume (pRBCs, plasma, platelets, and WB)

*OR* odds ratio, *CI* confidence interval, *GEE* generalized estimating equation, *sBP* systolic blood pressure, *HR* heart rate, *GCS* Glasgow coma scale, *AIS* abbreviated injury scale, *ISS* injury severity score, *TV* transfusion volume, *pRBC* packed red blood cell, *WB* whole blood

**Fig. 2:**
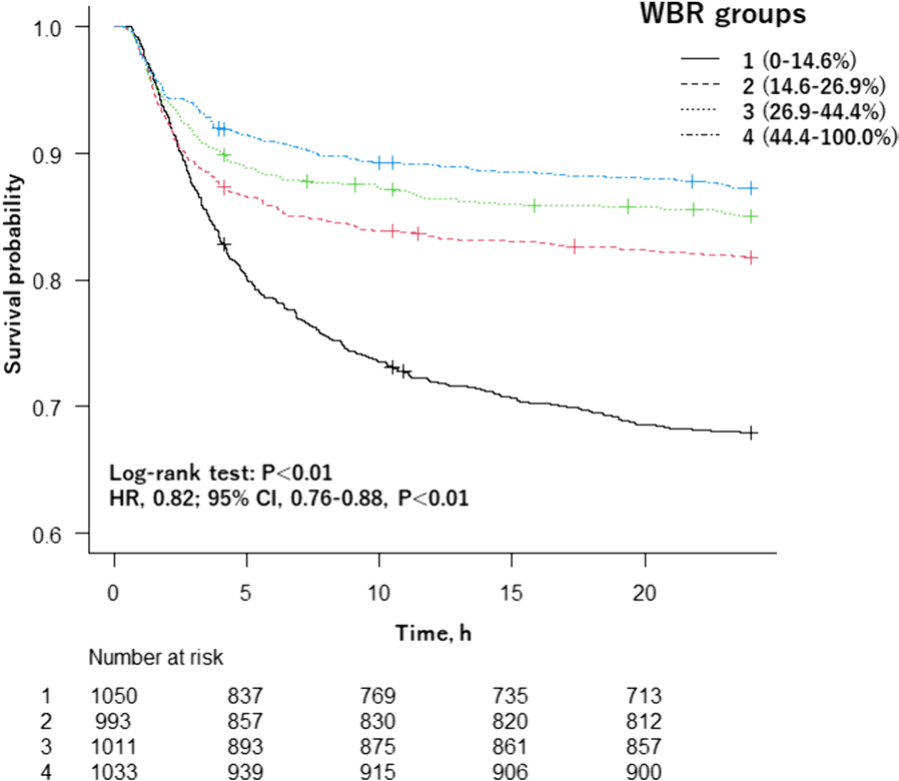
Kaplan–Meier 24-h survival curve. *WBR* whole blood ratio

**Fig. 3:**
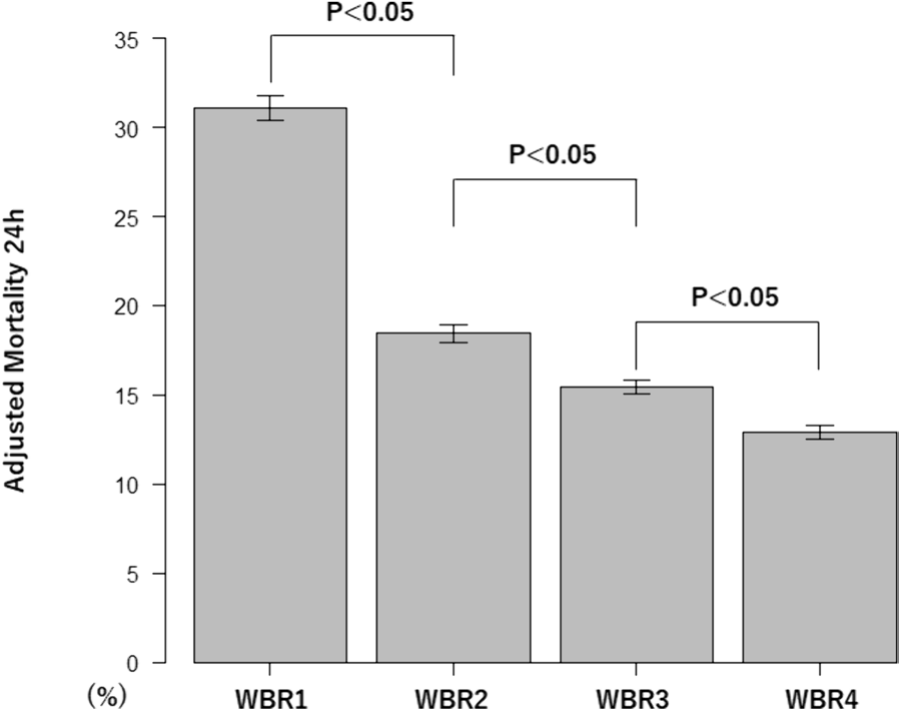
Adjusted rates of mortality 24-h between *WBR* groups WBR, whole blood ratio

**Fig. 4:**
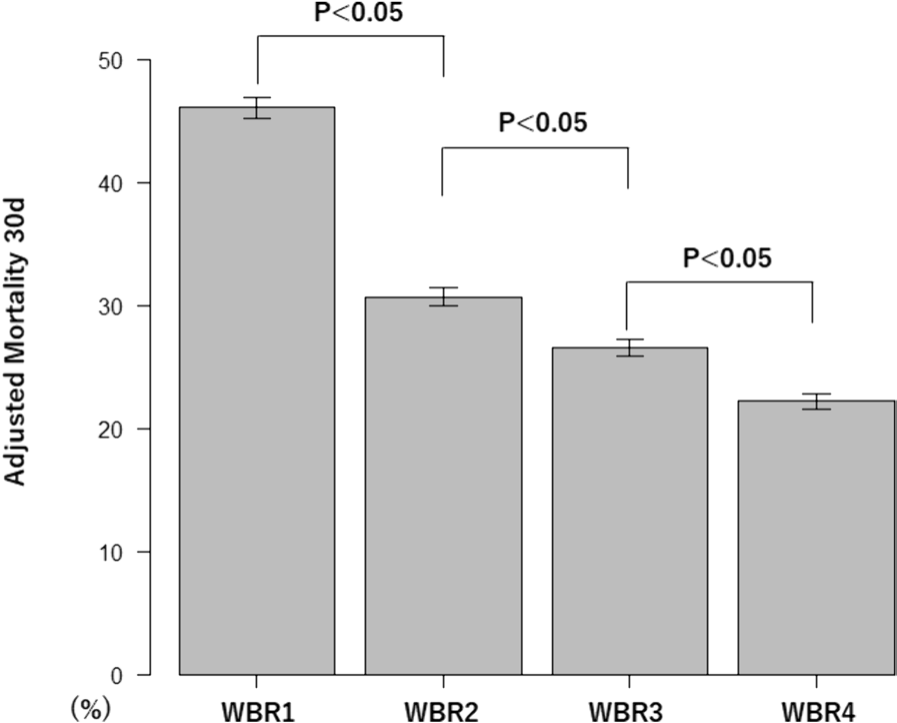
Adjusted rates of mortality 30-day between WBR groups. *WBR* whole blood ratio

Kaplan–Meier survival analysis with the log-rank test showed that the rate of 24-h mortality was significantly different between four WBR categories (Fig. 2). The log-rank trend test indicated that there is a decreasing trend on the rate of 24-h mortality from WBR1 to WBR4 (*P* < 0.01). Multivariate cox model showed WBR was associated with decreased 24-h mortality (HR, 0.82; 95% CI = 0.76–0.88) (Fig. 2). Figures 3 and 4 show the adjusted mortality 24-h and 30-day between WBR groups. The adjusted mortalities of 24-h and 30-day mortality also consistently decreased from WBR1 to WBR4.

The multivariable analysis showed that WBR was associated with lower TBV as well as lower odds of AKI (Supplementary Tables 4 and 5).

## Discussion

This study seemed to show that higher WBR was significantly associated with improved survival in trauma patients who received massive blood transfusion. Of note, the probability of mortality consistently decreased with higher WBR. Moreover, higher WBR was associated with reduced total blood transfusion volume and a lower incidence of AKI.

To date, only a few studies have reported the association between WBR and patient outcomes in trauma. A single centered prospective study reported that the ratio of WB to the total transfusion volume of component products was an independent predictor of survival [17]. Similarly, multicentered retrospective study in the military setting reported that a high ratio of WB to combined WB and PRBCs (> 33%) was associated with improved mortality [13]. Nevertheless, given other blood products, such as plasma and platelets, were administered in a balanced ratio, the strength of the current study lies in its focus on the case requiring massive transfusion in which transfusion strategy is align with the current trauma resuscitation strategy.

Potential mechanisms for improved outcomes related to the use of WB include provision of higher concentrations of clotting factors, improvement of hemostatic profile, lower transfusion requirement, and reversal of trauma-induced endotheliopathy [5]. A unit of WB contains higher concentration of red blood cells, plasma proteins, fibrinogen, and platelets compared to an equivalent unit of reconstituted blood. Previous literature suggested that WB transfusions were associated with faster resolution of shock [18], reduced transfusion volume [6, 19, 20], and attenuation of endothelial injury [21, 22]. In this study, increased WBR was associated with reduced total blood transfusion volume. Reconstituted whole blood by component blood transfusion had more additive solution and comparable dilutive solution [23]. Avoiding over-transfusion by using WB may have contributed to a lower incidence of AKI [24]. Additionally, the rate of hemostatic procedures was significantly lower in the study groups with higher WBR, suggesting that more liberal use of WBR may have contributed to earlier hemostasis.

The clinical implication of this study is that the resuscitation of patients undergoing massive blood transfusion for hemorrhagic shock may be improved by continuously administering WB. In this study, the median total transfusion volume was 4940 ml (IQR 3350–8504), and the potential benefit of WBR increase may be particularly significant in this cohort. While the safety profile of large-volume transfusion with WB remains unclear, a recent study suggested that the volume of WB similar to the current study (median 6.5 units [IQR 3–11]) was deemed safe and effective [25]. The cost-effectiveness of WB resuscitation has also been reported [26], ensuring that sufficient storage of WB at each trauma center might become the future trend.

## Limitations

There are several limitations to this study. First, patients in the WBR1 group (first quartile) were more severely injured than those in the other three WBR categories. While we created two multivariable logistic regression models to rigorously adjust for potential confounders, it is possible that there might have been other unadjusted confounding factors. To mitigate the risk of potential biases, we performed sensitivity analysis by excluding the WBR1 group which confirmed the association between higher WBR and improved mortality. Second, the cause of death was not captured in each case, given the limited information available in the TQIP database. Although we hypothesized the survival benefit of WBR in the context of hemorrhagic shock as hemorrhage is the most common cause of early trauma mortality [27], the use of WB might have influenced on the outcome of patients with other injury patterns such as traumatic brain injury. Third, while it has been reported that there is significant institutional variability in the use of WB transfusions. We evaluated the timing of administering WB and the quantities of blood products transfused within 4 h. In TQIP database, the quantities of blood products transfused within 24 h are not recorded. The most commonly used definition of massive transfusion is more than 10 units of red blood cells within 24 h [8], however, the definition has been recently shifted within shorter time frames and recent Delphi study noted a lower transfusion volume over a shorter time period could be suitable [28], and we thought our definition is acceptable. Fourth, the important covariate of this study such as tranexamic acid was not gain from TQIP database and not adjusted. Fifth, the missingness on variables and outcomes may affect the result of this study. We performed multiple imputation and confirmed the result of this study was not altered. Finally, due to the retrospective nature of our study, the observed survival benefit associated with higher WBR can only be interpreted as association, not causation.

## Conclusions

Our results suggest that the use of WB in higher proportions is associated with improved probability of survival in trauma patients requiring massive blood transfusion. Liberal use of whole blood might be considered for the resuscitation of trauma patients requiring massive blood transfusion.

## Supplementary Information

Supplementary Material 1: Figure 1. The number of patients requiring massive blood transfusion at each trauma center

Supplementary Material 2.

Supplementary Material 3.

Supplementary Material 4.

Supplementary Material 5.

Supplementary Material 6.

## Data Availability

The datasets used in the current study are available from the authors upon reasonable request.
