# Supplementary figures and images for: Association between whole blood ratio and risk of mortality in massively transfused trauma patients: retrospective cohort study
Source: Crit Care. 2024 Jul 19;28:253. doi: 10.1186/s13054-024-05041-8 (PMC11264807; doi:10.1186/s13054-024-05041-8)

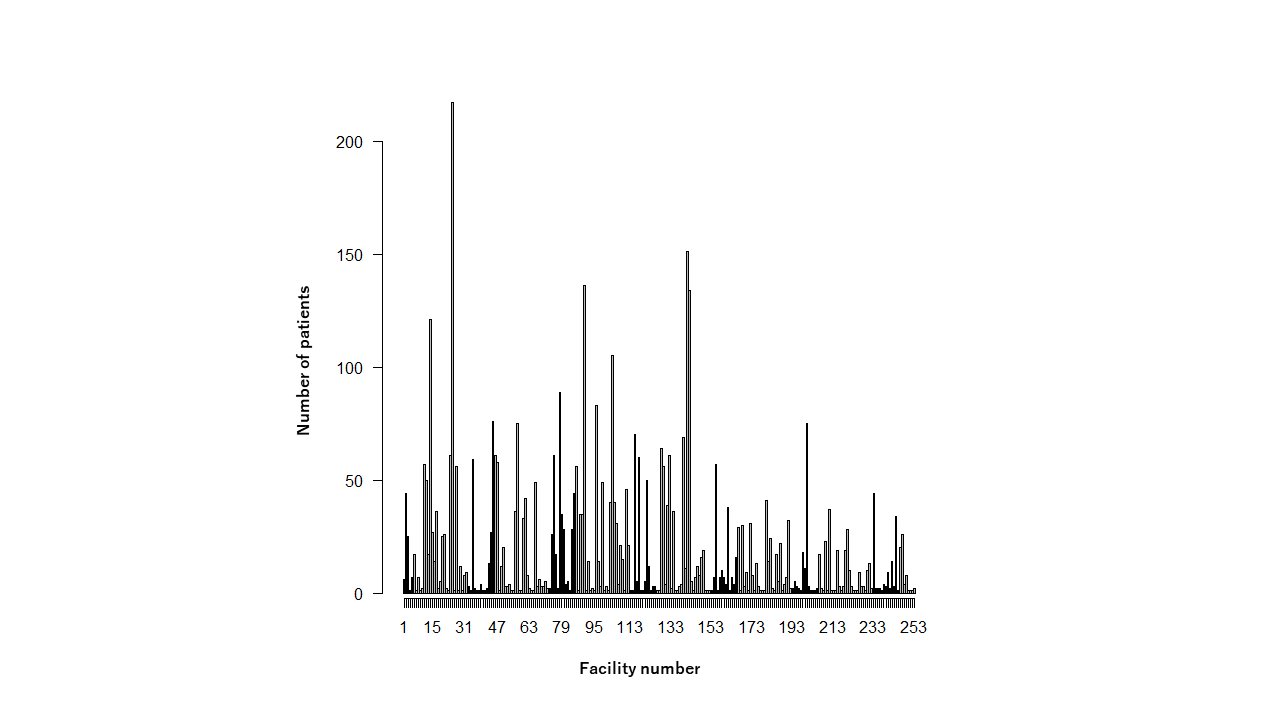

Supplement: Supplementary file 1 — Supplementary Material 1: Figure 1. The number of patients requiring massive blood transfusion at each trauma center [file 13054_2024_5041_MOESM1_ESM.tif]
