# Supplementary material for: Association between whole blood ratio and risk of mortality in massively transfused trauma patients: retrospective cohort study
Source: Crit Care. 2024 Jul 19;28:253. doi: 10.1186/s13054-024-05041-8 (PMC11264807; doi:10.1186/s13054-024-05041-8)
Supplement: Supplementary file 3 — Supplementary Material 3. [file 13054_2024_5041_MOESM3_ESM.docx]

Supplementary Table 1. Full models of logistic regression analysis for primary outcome.

| Model | Model 1 | | | Model 2 | | |
| --- | --- | --- | --- | --- | --- | --- |
| Variable | Coefficient | OR (95%CI) | P-value | Coefficient | OR (95%CI) | P-value |
| Age, y | 0.007 | 1.01 (1.00-1.01) | 0.01 | 0.006 | 1.00 (1.00-1.01) | 0.04 |
| Sex, male | -0.007 | 0.99 (0.78-1.26) | 0.95 | -0.087 | 0.91 (0.72-1.17) | 0.77 |
| Penetrating | -0.281 | 0.75 (0.60-0.94) | 0.01 | -0.360 | 0.69 (0.55-0.88) | <0.01 |
| sBP, mmHg | <0.001 | 1.00 (0.99-1.00) | 0.75 | <0.001 | 1.00 (0.99-1.00) | 0.77 |
| HR, beats per minute | -0.004 | 0.99 (0.99-0.99) | <0.01 | -0.004 | 0.99 (0.99-0.99) | <0.01 |
| GCS | -0.136 | 0.87 (0.85-0.88) | <0.01 | -0.127 | 0.88 (0.86-0.89) | <0.01 |
| AIS for head | -0.014 | 0.98 (0.92-1.05) | 0.63 | 0.022 | 1.02 (0.96-1.09) | 0.48 |
| AIS for chest | -0.023 | 0.97 (0.91-1.05) | 0.51 | -0.021 | 0.97 (0.91-1.05) | 0.55 |
| AIS for abdomen | 0.084 | 1.09 (1.01-1.17) | 0.02 | 0.042 | 1.04 (0.96-1.12) | 0.27 |
| AIS for peripheral | -0.167 | 0.84 (0.79-0.90) | <0.01 | -0.198 | 0.82 (0.76-0.87) | <0.01 |
| ISS | 0.003 | 1.00 (0.99-1.01) | 0.51 | 0.001 | 1.00 (0.99-1.01) | 0.83 |
| Timing of WB, min | <0.001 | 1.00 (1.00-1.00) | 0.18 | <0.001 | 1.00 (1.00-1.00) | 0.90 |
| Thoracotomy | 1.471 | 4.35 (3.29-5.75) | <0.01 | 1.330 | 3.78 (2.83-5.05) | <0.01 |
| Laparotomy | 0.304 | 1.36 (1.06-1.74) | 0.01 | 0.167 | 1.18 (0.91-1.53) | 0.20 |
| Level-1 | 0.290 | 1.34 (0.93-1.91) | 0.11 | 0.340 | 1.41 (0.96-2.05) | 0.07 |
| University affiliation | -0.120 | 0.88 (0.70-1.11) | 0.30 | -0.114 | 0.89 (0.70-1.13) | 0.34 |
| TBV, l | N.A | N.A | N.A | 0.096 | 1.10 (1.08-1.12) | <0.01 |

OR, odds ratio; CI, confidence interval; sBP, systolic blood pressure; HR, heart rate; GCS, Glasgow Coma Scale; AIS, abbreviated injury scale; ISS, injury severity score; TBV, total blood transfusion volume; N.A, not applicable
