# Supplementary material for: Association between whole blood ratio and risk of mortality in massively transfused trauma patients: retrospective cohort study
Source: Crit Care. 2024 Jul 19;28:253. doi: 10.1186/s13054-024-05041-8 (PMC11264807; doi:10.1186/s13054-024-05041-8)
Supplement: Supplementary file 4 — Supplementary Material 4. [file 13054_2024_5041_MOESM4_ESM.docx]

Supplementary Table 2. Sensitivity Analyses of Primary Outcomes in Whole Blood Ratio Groups Divided by Interquartile Range by Excluding WBR Group 1.

|  | WBR group | | |
| --- | --- | --- | --- |
| WBR group  IQR  n | WBR2  14.6-26.9%  n=993 | WBR3  26.9-44.4%  n=1,011 | WBR4  44.4-100%  n=1,033 |
| 24-hour mortality | 181 (18.2) | 151 (14.9) | 131 (12.7) |
| Odds ratio (95% CI) |  |  |  |
| Crude | Reference | 0.78 (0.62-0.99) | 0.65 (0.51-0.83) |
| GEE model 1 | Reference | 0.75 (0.57-0.98) | 0.58 (0.44-0.76) |
| GEE model 2 | Reference | 0.91 (0.69-1.20) | 0.76 (0.56-1.03) |

GEE model 1 was adjusted for age, sex, type of penetrating injury, sBP, HR, GCS, AIS for head, chest, abdomen, and peripheral injuries, ISS, timing of WB administration, thoracotomy, laparotomy, trauma center level, and university affiliation

GEE model 2 was adjusted for age, sex, type of penetrating injury, sBP, HR, GCS, AIS for head, chest, abdomen, and peripheral injuries, ISS, timing of WB administration, thoracotomy, laparotomy, trauma center level, university affiliation, and total transfusion volume (pRBCs, plasma, platelets, and WB)
