# Supplementary material for: Association between whole blood ratio and risk of mortality in massively transfused trauma patients: retrospective cohort study
Source: Crit Care. 2024 Jul 19;28:253. doi: 10.1186/s13054-024-05041-8 (PMC11264807; doi:10.1186/s13054-024-05041-8)
Supplement: Supplementary file 5 — Supplementary Material 5. [file 13054_2024_5041_MOESM5_ESM.docx]

Supplementary Table 3. Multivariable Analysis of Continuous Secondary Outcomes Based on Whole Blood Ratio Category

| Continuous outcome | β | 95% Confidence interval |
| --- | --- | --- |
| TBV, liters | -10.7 | -11.3 to -10.0 |
| ICU LOS, days | 0.03 | -0.31 to 0.38 |

Models were adjusted for age, sex, type of penetrating injury, sBP, HR, GCS, AIS for head, chest, abdomen, and peripheral injuries, ISS, timing of WB administration, thoracotomy, laparotomy, trauma center level and university affiliation

TBV, total blood transfusion volume; ICU, intensive care unit; LOS, length of hospital stays; sBP, systolic blood pressure; HR, heart rate; GCS, Glasgow Coma Scale; AIS, abbreviated injury scale; ISS, injury severity score
