# Supplementary material for: Association between whole blood ratio and risk of mortality in massively transfused trauma patients: retrospective cohort study
Source: Crit Care. 2024 Jul 19;28:253. doi: 10.1186/s13054-024-05041-8 (PMC11264807; doi:10.1186/s13054-024-05041-8)
Supplement: Supplementary file 6 — Supplementary Material 6. [file 13054_2024_5041_MOESM6_ESM.docx]

Supplementary Table 4. Multivariable Analysis of Binary Secondary Outcomes Based on Whole Blood Ratio Category

| Outcome | Adjusted odds ratio | 95% Confidence interval |
| --- | --- | --- |
| complications^*^  AKI  DVT  PE  ARDs  Stroke  MI | 0.71  1.07  1.19  0.96  0.90  0.86 | 0.63-0.80  0.95-1.21  1.02-1.38  0.79-1.16  0.73-1.12  0.57-1.28 |

＊Exclusion of mortality within 24 hours.

Models were adjusted for age, sex, type of penetrating injury, sBP, HR, GCS, AIS for head, chest, abdomen, and peripheral injuries, ISS, timing of WB administration, thoracotomy, laparotomy, trauma center level, and university affiliation

AKI, acute kidney injury; DVT, deep vein thrombosis; PE, pulmonary embolism; ARDs, acute respiratory distress syndrome; MI, myocardial infarction; sBP, systolic blood pressure; HR, heart rate; GCS, Glasgow Coma Scale; AIS, abbreviated injury scale; ISS, injury severity score
